# A constitutive model for the time-dependent, nonlinear stress response of fibrin networks

**DOI:** 10.1007/s10237-015-0649-1

**Published:** 2015-01-25

**Authors:** Thomas H. S. van Kempen, Gerrit W. M. Peters, Frans N. van de Vosse

**Affiliations:** 1 Department of Biomedical Engineering, Eindhoven University of Technology, PO Box 513, 5600MB Eindhoven, The Netherlands; 2 Department of Mechanical Engineering, Eindhoven University of Technology, Eindhoven, The Netherlands

**Keywords:** Blood clotting, Constitutive modeling, Large amplitude oscillatory shear (LAOS), Biopolymer networks, Sensitivity analysis

## Abstract

Blood clot formation is important to prevent blood loss in case of a vascular injury but disastrous when it occludes the vessel. As the mechanical properties of the clot are reported to be related to many diseases, it is important to have a good understanding of their characteristics. In this study, a constitutive model is presented that describes the nonlinear viscoelastic properties of the fibrin network, the main structural component of blood clots. The model is developed using results of experiments in which the fibrin network is subjected to a large amplitude oscillatory shear (LAOS) deformation. The results show three dominating nonlinear features: softening over multiple deformation cycles, strain stiffening and increasing viscous dissipation during a deformation cycle. These features are incorporated in a constitutive model based on the Kelvin–Voigt model. A network state parameter is introduced that takes into account the influence of the deformation history of the network. Furthermore, in the period following the LAOS deformation, the stiffness of the networks increases which is also incorporated in the model. The influence of cross-links created by factor XIII is investigated by comparing fibrin networks that have polymerized for 1 and 2 h. A sensitivity analysis provides insights into the influence of the eight fit parameters. The model developed is able to describe the rich, time-dependent, nonlinear behavior of the fibrin network. The model is relatively simple which makes it suitable for computational simulations of blood clot formation and is general enough to be used for other materials showing similar behavior.

## Introduction

Blood clots form to prevent blood loss after a vascular injury but can also lead to undesired complications such as thrombosis when forming intravascularly. The mechanical properties of the blood clot are of major of importance for its functioning and have been related to many diseases (Weisel 2008) but remain elusive to model. The main structural component of the blood clot is fibrin, a fibrous network that forms within the blood clot and thereby provides strength to the platelet plug that forms as a provisional closure of the injury. The fibrin network shows rich nonlinear mechanical behavior that enables the network to perform its physiological function (Brown et al. 2009; Münster et al. 2013), but this behavior is not fully described by current constitutive models. Therefore, in this study, a constitutive model is developed for the nonlinear mechanical behavior of the fibrin network.

The fibrin network forms in multiple steps after the conversion of fibrinogen to fibrin monomers, enzymatically catalyzed by thrombin. These monomers aggregate to form two-stranded structures known as protofibrils which subsequently polymerize into fibers that eventually form the fibrin network (Cilia La Corte et al. 2011). This network is furthermore strengthened due to the presence of factor XIII (fXIIIa) that creates cross-links within and between protofibrils (Ryan et al. 1999; Lorand 2005). It is the hierarchical structure of the fibers that gives the fibrin network its remarkable, yet complicated, mechanical properties (Brown et al. 2009; Piechocka et al. 2010; Münster et al. 2013). One of the most pronounced nonlinear mechanical effects is that fibrin stiffens with an increasing deformation (strain stiffening) (Shah and Janmey 1997; Brown et al. 2009; Kang et al. 2009; Weigandt et al. 2011; Münster et al. 2013). During such a deformation, individual fibers can stretch to multiple times their own length (Liu et al. 2006). Upon repeated deformation cycles, the fibers persistently lengthen, leading to a lower stiffness at the same strain and hence a softening effect (Münster et al. 2013). The combination of these nonlinear viscoelastic features makes it complicated to describe the mechanical properties of the fibrin network, yet they are essential for a realistic description of the mechanical (in-situ) behavior where complex loading histories may occur. Therefore, the goal of this study is to develop a constitutive model for the nonlinear viscoelastic, thixotropic behavior of the fibrin network. To our knowledge, such a model, suitable for advanced numerical simulations of blood clot formation (e.g., Storti et al. 2014) and based on a continuum mechanics approach, has not been developed before.

The nonlinear mechanical properties of fibrin, and networks of biopolymers in general, have been studied using models and various experimental techniques. Models have been used to show that the strain stiffening behavior can have an entropic or a nonaffine origin (Storm et al. 2005; Onck et al. 2005), while experimentally, it has been shown that both mechanisms play a role, most likely at different strain regimes (Brown et al. 2009; Piechocka et al. 2010; Weigandt et al. 2011). The intrinsic nonlinear behavior of single fibrin fibers has been studied (Liu et al. 2006; Averett et al. 2012) as well as its influence on network mechanics (Hudson et al. 2010). Recently, it has been shown that parts of fibrin fibers can relocate within the network which makes it a dynamic structure that can remodel (Chernysh et al. 2012).

Various experimental protocols have been used to probe nonlinear mechanical properties of fibrin and other biopolymers, including strain ramps (Schmoller et al. 2010), compression (Kim et al. 2014), differential prestress (Piechocka et al. 2010) and large amplitude oscillatory shear (LAOS) (Münster et al. 2013). Each protocol probes different aspects of the nonlinear viscoelastic behavior of the material and a combination is useful to obtain a complete description (Semmrich et al. 2008; Broedersz et al. 2010). In this study, a LAOS deformation is used to develop a constitutive model for the nonlinear viscoelastic behavior of the fibrin network. An advantage of LAOS is that it is suitable to probe the rich nonlinear viscoelastic behavior of a material, while it is still possible to distinguish the various features observed. The repeated oscillatory deformation provides insights into the thixotropic behavior of the fibrin network. Furthermore, the LAOS deformation is a straightforward extension of the small amplitude oscillatory shear (SAOS) deformation that is usually used to study viscoelastic behavior. Also, the response of the fibrin network to a large oscillatory deformation mimics the physiological deformation due to an oscillatory blood flow that these networks have to withstand. This makes LAOS ideally suited for the development of a constitutive model of the fibrin network. LAOS deformations have been used before to study the nonlinear viscoelastic properties of fibrin (Münster et al. 2013). In this study, the experimental data are used to unravel the viscoelastic response of the fibrin networks and develop a constitutive model.

In the next section, experiments are introduced, the results of which are used subsequently to develop the constitutive model. The model is then used to describe and predict the results of the experiments, followed by a discussion of the outcome.

## Experimental methods

### Fibrin network formation

Fibrin networks are formed by adding 0.5 U/ml human thrombin to 1 mg/ml human fibrinogen (Kordia, Leiden, The Netherlands) after which the sample is quickly transferred to the titanium cone-plate geometry (25 mm diameter, 0.02 rad cone angle) of an ARES rheometer (Rheometric Scientific, USA). To follow the network formation, a SAOS deformation with a frequency of 1 Hz and strain amplitude of 0.01 is imposed for 2 h. This deformation is within the linear viscoelastic regime and does not interfere with the network formation (van Kempen et al. 2014). The networks are formed at $37\,^{\circ }\mathrm {C}$ and a layer of mineral oil is applied to the sample edge to minimize evaporation. The response of the networks during the SAOS deformation is predominantly elastic (van Kempen et al. 2014), indicated by an elastic modulus $G^{\prime }$ that exceeds the viscous modulus $G^{\prime \prime }$ many times. Therefore, the SAOS data are presented in terms of the elastic modulus only.

### Large amplitude oscillatory shear (LAOS) experiment

**Fig. 1:**
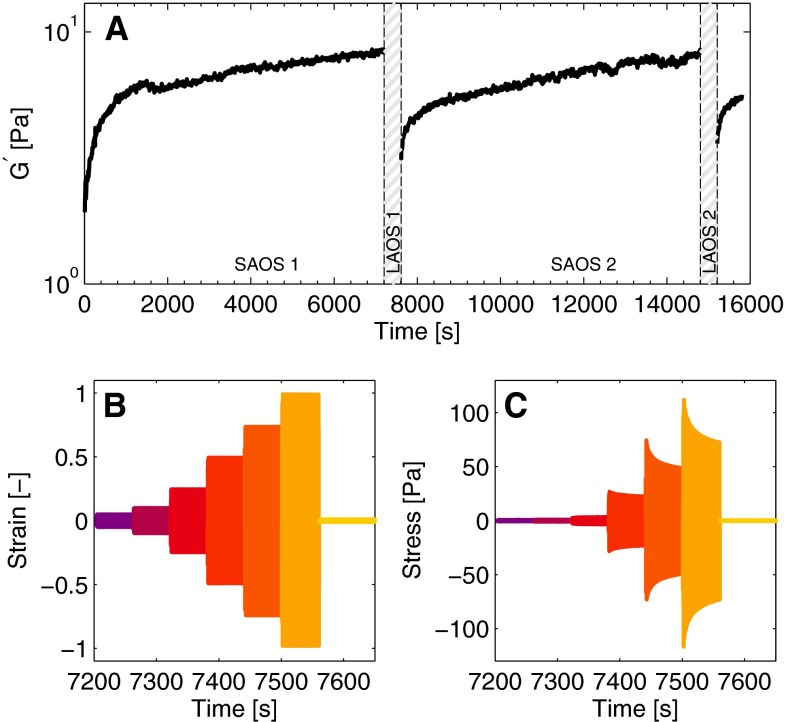
The elastic modulus, $G^{\prime }$, in time is measured during a SAOS experiment of 2 h (**a**). Then, a LAOS experiment is performed in which the strain amplitude is increased (**b**), and the resulting stress is measured (**c**). This procedure is repeated afterward

After the network formation, the nonlinear viscoelastic properties are studied by imposing a LAOS deformation. While the frequency of the oscillatory deformation is always maintained at $1$ Hz, the strain amplitude is increased in discrete steps of 60 s to 0.05, 0.1, 0.25, 0.5, 0.75 and 1.0. After the LAOS deformation, the response of the network is followed for 2 h by imposing the same SAOS deformation as before. Subsequently, the LAOS sequence is repeated. The first LAOS sequence is used to determine parameter values of the different parts of the constitutive model, as explained later. The second sequence is used to test the predicting capabilities of the model. An overview of this protocol is shown in Fig. 1. For convenience, in the remainder of this paper, the start of a LAOS sequence is defined as starting time i.e., $t=0$.

The raw data from the rotation and torque signals are collected using an analog-to-digital converter (ADC) (Wilhelm 2002) and converted to strain and stress. The data obtained in this way during the SAOS experiment contain a large amount of noise, due to the low torque. Therefore, the strain and stress during the SAOS experiment are not obtained from the raw data but using the data provided by the rheometer software. These data, provided in terms of strain amplitude and the elastic and viscous modulus, are used to reconstruct the strain and stress signals in time.

The data are analyzed in terms of Lissajous–Bowditch plots in which the strain is plotted versus the stress, showing closed loops that illustrate the nonlinear viscoelastic behavior of the fibrin network (Ewoldt et al. 2008; Hyun et al. 2011). Different aspects of these plots are used as a guideline for developing the constitutive model.

To examine the influence of cross-links formed by fXIIIa, present in the fibrinogen stock solution as shown by SDS-PAGE, some of the samples are allowed to polymerize for one instead of 2 h. Since the cross-linking occurs over a relatively long time scale compared with the network formation itself, a network polymerized for 1 h has less cross-links (Ryan et al. 1999; Lorand 2005). It is expected that this will influence the nonlinear viscoelastic behavior (Münster et al. 2013).

## Model development

The constitutive model is an extension of a Kelvin–Voigt model, a relatively simple model for a viscoelastic solid (Barnes et al. 1989). In a previous study, the model has been used to describe the maturation of the fibrin network (van Kempen et al. 2014). The model relates the shear stress $\tau$ to the strain $\gamma$ and its temporal derivative, the strain rate $\dot{\gamma }$, as

1 $$\begin{aligned} \tau = G \, \gamma + \eta \, \dot{\gamma }. \end{aligned}$$

The shear modulus $G$ and viscosity $\eta$ have been related to structural quantities of the network during its maturation (van Kempen et al. 2014). Although this connection to structural quantities is still useful, this relation is not used in this study explicitly. Instead, the shear modulus and viscosity become dependent on the strain history, $G = G\left( \gamma , t\right)$ and $\eta = \eta \left( \gamma , t\right)$. In this way, the Kelvin–Voigt model is extended by including the nonlinear features that are observed in the results of the LAOS experiments.

**Fig. 2:**
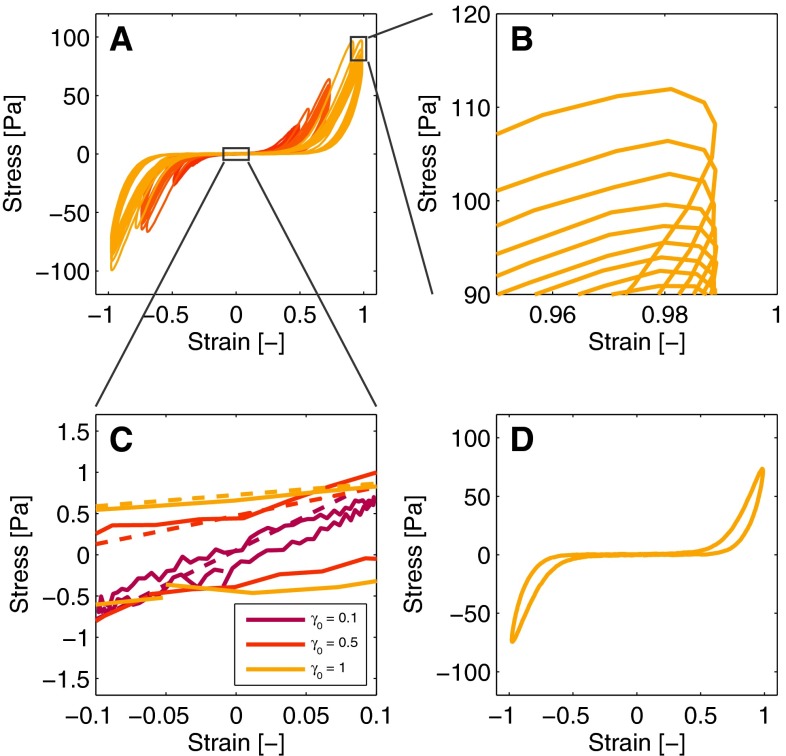
The Lissajous–Bowditch plots shown in *panel a* illustrate the nonlinear behavior (**a**). Note that every fifth cycle is plotted for clarity. Zooming in on the maximal stress values illustrates that the stress decreases over multiple deformation cycles (**b**). Zooming in on the origin illustrates the same effect (**c**). The slopes of the *dashed lines* in *panel c* correspond to the estimated minimal strain modulus, $G_0$. The increasing viscous dissipation during the deformation cycle is illustrated by the observation of a single loop (**d**). The *colors* correspond to the strains shown in Fig. 1b

Representative results of a LAOS experiment, illustrating the nonlinear response of the fibrin network, are shown in Fig. 2 and subsequently used to explain the development of the model.

### Nonlinear features and underlying assumptions

The Lissajous–Bowditch plots shown in Fig. 2a clearly deviate from an ellipse, indicating the nonlinear viscoelastic behavior of the fibrin network. Three nonlinear features can be distinguished by observing the plots in detail. The first feature is a cycle-dependent softening effect that takes place over multiple deformation cycles. This is visible as a decrease in the stress at maximal strain (Fig. 2b) and also as a decrease in the slope of the curve at minimum strain (Fig. 2c). The second feature is strain stiffening, illustrated by the increased slope of the curves with increasing strain (Fig. 2d). The third feature is that the viscous dissipation increases with increasing strain, shown by the broadening of the cycles throughout the cycle (Fig. 2d).

Besides these nonlinear phenomena observed during the LAOS experiment, a fourth phenomenon observed is that the elastic modulus increases during the 2 h after the LAOS experiment (Fig. 1a). This increase is attributed partially to recovery of the network after the large deformation and to the continuous creation of new cross-links due to fXIIIa (Ryan et al. 1999; Lorand 2005). This effect, and the three nonlinear features are incorporated in the Kelvin-Voigt model. The focus is on the description of the mechanical properties of the fibrin network during the first LAOS experiment and to a lesser extent on the response during the second LAOS experiment.

#### Softening and subsequent recovery

As shown in Fig. 2b, c, the stiffness of the fibrin network decreases during the multiple deformation cycles of the LAOS experiment. This effect is attributed to the lengthening of fibers due to the increasing cyclic deformation that leads to a lower stiffness (Münster et al. 2013). When subsequently the amplitude of the strain is decreased, the stiffness of the network rises, as shown by the increasing $G^{\prime }$ value in Fig. 1a. Both effects are included in the model using a network state parameter (NSP), $x$, that describes the change in mechanical properties of the fibrin network over time based on its deformation history. This is simply modeled by making the low-strain shear modulus proportional to the NSP,

2 $$\begin{aligned} G_0 = G_{00}\,x, \end{aligned}$$

with $G_{0}$ the modulus at low strain and $G_{00}$ the low-strain modulus of the virgin network, which is the stiffness at a reference state, chosen as the start of the first LAOS experiment. The form of Eq. (2) implies that the NSP is always nonnegative and at the reference state equal to one. More complicated expressions can be used if desired but a linear relation is used here as a convenient start to limit the complexity of the model. In the following, an evolution equation for $x$ is developed based on the behavior of $G_0$, but it is first outlined how this behavior is extracted from the experimental data.

Although the softening effect that takes place during multiple deformation cycles is best illustrated by observing the stress values at maximal strain ($\tau _0$) as shown in Fig. 2b, this effect influences the stress throughout the entire deformation cycle. To quantify the softening, values of $\tau _0$ could be used, but a drawback is that the stress at maximal strain is influenced by both the softening effect and strain stiffening. Therefore, the modulus at minimal strain, $G_0$, is used (see Fig. 2c) to be able to quantify the kinetics of the softening from the data, independent of the other nonlinear effects.

The values of $G_0$ can be interpreted as a local derivative of the stress with respect to strain, at the strain $\gamma = 0$,

3 $$\begin{aligned} G_0\left( t,\gamma \right) = \left. \frac{\partial \tau }{\partial \gamma }\right| _{\gamma = 0}, \end{aligned}$$

and are estimated by a linearization of the positive stresses at the strains of $\gamma = \pm 0.05$. For the cycles with a strain amplitude of $0.01$, the stresses at maximal strain are used for the linearization. The accuracy of this method increases with increasing strain amplitude, because the corresponding stresses are higher and the signals contain less noise, as visible in Fig. 2c. Values for the NSP are subsequently found using the relation between the shear modulus and the NSP (Eq. (2)),

4 $$\begin{aligned} x\left( t, \gamma \right) = \frac{G_{0}}{G_{00}}. \end{aligned}$$

**Fig. 3:**
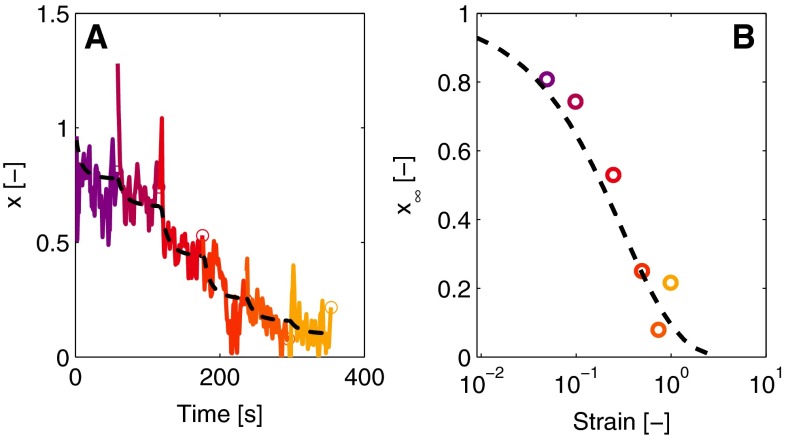
The NSP, $x$, in time during LAOS (**a**). The *colors* correspond to the strains shown in Fig. 1b. After a change in strain, the NSP levels off to a new value denoted by $x_\infty$ that decreases with the strain (**b**). The *dashed lines* are obtained with the model [see Eqs. (5) and (6)]

Results for the NSP during a LAOS experiment are shown in Fig. 3 with the colors corresponding to the LAOS protocol in Fig. 1b.

Note that this implies that the NSP obtained in this way is intrinsically noisy since it is determined using the stress at the lowest possible strain. This especially plays a role during the lowest strains applied (Fig. 3a).

As shown in Fig. 3a, the NSP decreases due to the applied strain, which should be described by the evolution equation for $x$. After increasing the strain, the NSP decreases toward a value that depends on the corresponding strain amplitude. The value to which the NSP levels off is referred to as $x_{\infty }$, and the decrease toward this value goes with a time constant $c_{d}$. As observed during the SAOS deformation imposed after the LAOS sequence, the stiffness of the network rises again when the deformation amplitude is decreased (see Fig. 1a). This means that $x$ increases, in principle to the $x_{\infty }$ value related to that lower strain. This increase is modeled with a time constant $c_{i}$. The above thus leads to an evolution equation for $x$,

5 $$\begin{aligned} \dot{x} = {\left\{ \begin{array}{ll} -c_{d}\,\left( x - x_{\infty }\right) &{} x>x_{\infty },\\ -c_{i}\,\left( x - x_{\infty }\right) &{} x\le x_{\infty }. \end{array}\right. } \end{aligned}$$

The values of $x_{\infty }$ at which the NSP levels off are related to the imposed strain, $x_{\infty } = x_{\infty }\left( \gamma \left( t\right) \right)$. An expression for this function is found by observing the values of the NSP at the end of each strain step during the LAOS protocol, illustrated by the circles in Fig. 3b. These values are not necessarily the steady-state value that $x$ would reach if the strain amplitude is imposed for a longer period than $60$ s. Nevertheless, they provide useful information about the expression that the equation for $x_{\infty }$ should have. Requirements are that the expression is non-negative, independent of the direction of strain and equal to one at minimal strain. A suitable expression for $x_{\infty }$ is,

6 $$\begin{aligned} x_{\infty } = e^{-a\left| \gamma \right| ^b}, \end{aligned}$$

with $a$ and $b$ fit parameters. The expression for $x_{\infty }$ is shown in Fig. 3b together with experimental results.

The evolution equation for $x$ fitted to the experimental data is shown in Fig. 3a.

#### Strain stiffening

**Fig. 4:**
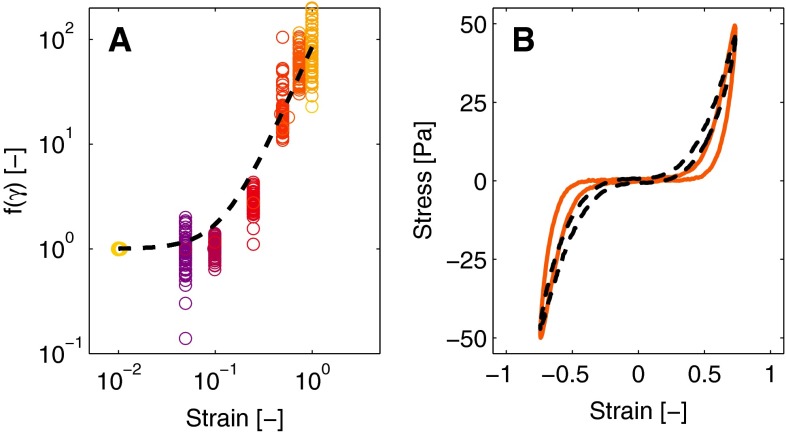
The function that describes the increase of the stiffness as function of the strain (**a**). The experimental values are shown in *colored circles*, and the model description with a *dashed line*. *Panel b* shows a single loop illustrating the nonlinear viscous dissipation as measured (*colored line*) and the modeled (*dashed line*)

The stiffness of the fibrin network increases with strain during a deformation cycle. Several physical explanations can be given for this strain stiffening behavior, such as entropic stretching (Storm et al. 2005), non-affine deformations (Onck et al. 2005) and protein unfolding (Brown et al. 2009). In this study, the strain stiffening is incorporated in the model in a phenomenological way by making the shear modulus $G$ a function of the strain $\gamma$. To obtain an expression based on the experimental data, the values of the stress at maximal strain during each deformation cycle are used. When the strain reaches its maximal value during the deformation cycle, the strain rate is instantaneously zero and the viscous contribution in the Kelvin-Voigt model vanishes [see Eq. (1)]. Using Eqs. (1, 2), the stress is given by,

7 $$\begin{aligned} \tau _0 = G_{00}\,x\,f\left( \gamma _0\right) \,\gamma _0, \end{aligned}$$

with $\tau _0$ the stress at maximal strain $\gamma _0$ and $f\left( \gamma \right)$ a function of the strain that describes the strain stiffening behavior. Since all quantities, except $f\left( \gamma _0\right)$, are known, Eq. (7) can be rewritten to,

8 $$\begin{aligned} f\left( \gamma _0\right) = \frac{\tau _0}{G_{00}\,x\,\gamma _0}, \end{aligned}$$

to obtain an expression for $f\left( \gamma \right)$ (Fig. 4a).

The expression for $f\left( \gamma \right)$ should equal one at low strain, increase with increasing strain and be symmetric with respect to strain. Furthermore, it should be a function of the invariants of the deformation tensor (Macosko 1994). A function that satisfies all these criteria, and gives large flexibility with only two parameters, is the function,

9 $$\begin{aligned} f\left( \gamma \right) = \left( 1 + k_1 \, \gamma ^2\right) ^{n_1}\!\!, \end{aligned}$$

with $k_1$ and $n_1$ as fit parameters. As shown in Fig. 4a, this function describes the data reasonably well.

#### Nonlinear viscous dissipation

The viscous dissipation changes throughout a deformation cycle, illustrated by the broadening of the Lissajous–Bowditch plots with increasing strain (Fig. 4b). This feature is incorporated in the model by making the viscous contribution an increasing function of the strain,

10 $$\begin{aligned} \eta = \eta _0 \, g\left( \gamma \right) , \end{aligned}$$

with $\eta _0$ the viscosity at minimal strain, and $g\left( \gamma \right)$ the function describing the strain dependency. The same expression as used to describe the strain stiffening is chosen,

11 $$\begin{aligned} g\left( \gamma \right) = \left( 1 + k_2 \, \gamma ^2\right) ^{n_2}\!\!, \end{aligned}$$

with $k_2$ and $n_2$ fit parameters.

### Overview of the constitutive model

Combining the above, the constitutive model reads,

12 $$\begin{aligned}&\tau = G_{00}\, x \,\left( 1 + k_1 \, \gamma ^2\right) ^{n_1}\, \gamma + \eta _0 \,\left( 1 + k_2 \, \gamma ^2\right) ^{n_2}\, \dot{\gamma } \end{aligned}$$

13 $$\begin{aligned}&\dot{x} = {\left\{ \begin{array}{ll} -c_{d}\,\left( x - x_{\infty }\right) &{} x>x_{\infty },\\ -c_{i}\,\left( x - x_{\infty }\right) &{} x\le x_{\infty },\\ \end{array}\right. } \end{aligned}$$

14 $$\begin{aligned}&x_{\infty } = e^{-a\left| \gamma \right| ^b}, \end{aligned}$$

with $c_{d}$, $c_{i}$, $a$, $b$, $k_1$, $n_1$, $k_2$ and $n_2$ fitting parameters that are obtained from the data using a stepwise fitting procedure as explained next.

### Numerical procedures and parameter optimization

**Fig. 5:**
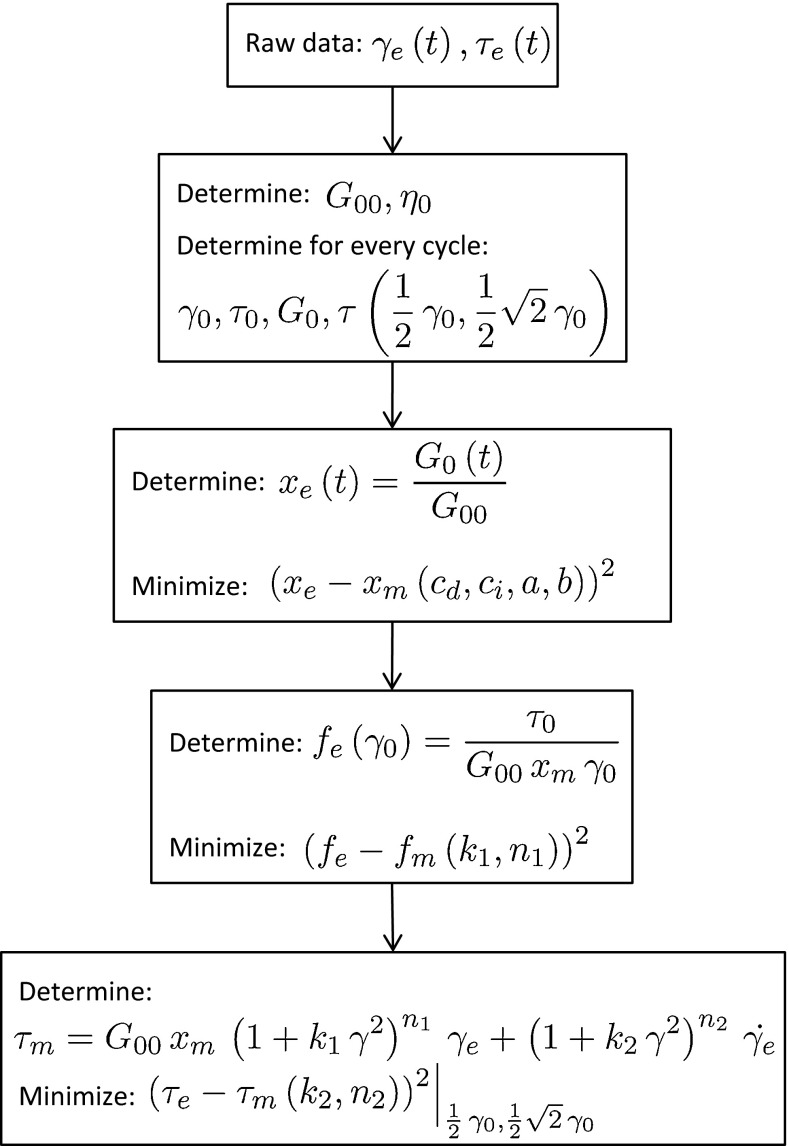
Flowchart of the parameter optimization process

The constitutive model contains eight fit parameters that are used to describe the nonlinear phenomena observed in the Lissajous–Bowditch plots. The evolution equation of the NSP, $x$, contains four parameters, $c_{d}$, $c_{i}$, $a$ and $b$. The function used to describe strain stiffening contains two parameters, $k_1$ and $n_1$, similarly to the function that describes the viscous dissipation, $k_2$ and $n_2$. These parameter sets are determined in three consecutive steps, based on the first LAOS sequence and the subsequent SAOS measurement of 2 h. This parameter set is then used to study the predicting capabilities of the model using the second LAOS sequence. A flow chart of the fitting procedure is shown in Fig. 5, where experimental values are denoted by a subscript $e$ and model values by $m$.

The strain and stress signals obtained during an experiment are analyzed using an algorithm implemented in MATLAB (The MathWorks, Natick, MA). First, values for the (linear) shear modulus $G_{00}$ and viscosity $\eta _0$ are obtained from the elastic and viscous modulus (van Kempen et al. 2014) using the data from the strain of $0.01$ at the beginning of the LAOS sequence. Subsequently, for every deformation cycle the maximum strain amplitude $\gamma _0$, the corresponding stress $\tau _0$, the zero strain modulus $G_{0}$ and the stresses at $0.5\,\gamma _0$ and $\frac{1}{2}\sqrt{2}\,\gamma _0$ are determined. The values of $G_0$ are used to determine the NSP from the experimental data, denoted by $x_e$, to which the evolution equation for the NSP is fit. The NSP describes the decrease of the modulus during the LAOS deformation and the recovery afterward. To avoid that the SAOS measurement of 2 h dominates the fitting procedure it is given a relative weight of $0.01$ compared with the six measurements of 60 s each of the LAOS sequence. In this way, values for the parameters $c_{d}$, $c_{i}$, $a$ and $b$ are obtained.

Using the obtained NSP and the values for $\tau _0$, $\gamma _0$ , the strain stiffening function $f_e\left( \gamma \right)$ is determined for every cycle and used to find the parameters $k_1$ and $n_1$ in a second fitting step. Finally, the parameters that describe the nonlinear viscous dissipation $k_2$ and $n_2$ are determined by calculating the stress at strains of $1/2\,\gamma _0$ and $\frac{1}{2}\sqrt{2}\,\gamma _0$ and comparing them with the respective experimental stresses. The strains of $1/2\,\gamma _0$ and $\frac{1}{2}\sqrt{2}\,\gamma _0$ are chosen because at those values the viscous contribution to the stress, and the stress itself, are relatively large.

The three fitting procedures are performed using the nonlinear least-squares solver *lsqnonlin* with a trust-region-reflective algorithm as implemented in the Global Optimization Toolbox of MATLAB. The fitting procedures are performed multiple times with initial parameter values chosen randomly from a broad interval using the Multistart algorithm. In this way, it is avoided that local minima are found.

### Sensitivity analysis

The constitutive model developed contains eight parameters that are estimated using experimental data. To get a deeper insight into the sensitivity of the model to these parameters, a sensitivity analysis is performed. A global variance-based method (Sobol 2001) is applied here, that considers the total output variance and determines the contribution of each model parameter to this variance by itself and through interactions with other parameters. These contributions are expressed as sensitivity indices that are estimated using Saltelli’s method (Saltelli 2002). A sensitivity index is the contribution of a parameter to the variance of that output, relative to the total variance of the output. For every output, two sensitivity indices for every parameter are defined.The main sensitivity index, $S_i$, quantifies the direct influence of parameter $i$ on the output, whereas the total sensitivity index, $S^T_i$, describes this main effect but in addition also the influence on the output due to all higher-order contributions in which parameter $i$ is involved (Huberts et al. 2014). The main index can be used to show which parameters are most rewarding to determine more accurately, while the total index shows which parameters could be fixed. More details about this method can be found elsewhere (Saltelli 2002; Huberts et al. 2014).

**Fig. 6:**
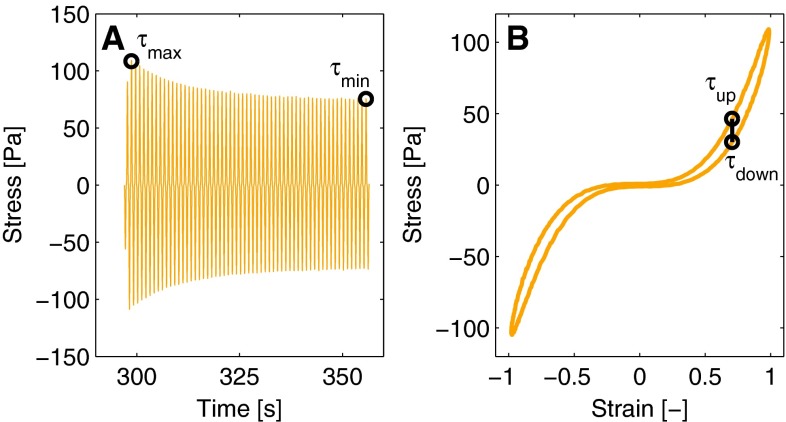
Illustration of the stresses that are used for the outputs of the sensitivity analysis. Softening is quantified using the decrease of the stress during the strain interval (**a**). Stiffening is quantified using the maximal stress $\tau _\mathrm{max}$. The output for the nonlinear viscous behavior is the difference between the stresses at the strain $\gamma = \frac{1}{2}\sqrt{2}\,\gamma _0$ (**b**)

The output quantities, on which the influence of the variance for each parameter is obtained, are inspired by the three main features observed in the nonlinear viscoelastic response of the fibrin network. The first output quantifies the softening effect, and is defined as the relative decrease of the stress at maximal strain during the strain interval with amplitude, $\gamma _0 = 1$,

15 $$\begin{aligned} O_\mathrm{so} = \frac{\tau _\mathrm{max} - \tau _\mathrm{min}}{\tau _\mathrm{max}}, \end{aligned}$$

with $\tau _\mathrm{max}$ and $\tau _\mathrm{min}$ the maximal stress during the first and last full cycle, respectively (see Fig. 6a).

Strain stiffening is quantified using the maximal stress during this interval, which occurs at the maximum strain amplitude of the first full cycle, $\tau _\mathrm{max}$. This value is normalized with the low-strain modulus of the virgin network $G_{00}$,

16 $$\begin{aligned} O_\mathrm{ss} = \frac{\tau _\mathrm{max}}{G_{00}}. \end{aligned}$$

The third output quantifies the nonlinear viscous dissipation and is defined as the difference between the stresses at $\gamma = \frac{1}{2}\sqrt{2}\,\gamma _0$ for the increasing and decreasing part of the first full cycle of the interval with $\gamma _0 = 1$,

17 $$\begin{aligned} O_\mathrm{vi} = \tau _\mathrm{up} - \tau _\mathrm{down}, \end{aligned}$$

with $\tau _\mathrm{up}$ and $\tau _\mathrm{down}$ the corresponding stresses as shown in Fig. 6b.

The outputs defined above are obtained using a parameter set drawn from a specified uncertainty range using Latin hypercube sampling (Saltelli 2002). The parameter range is based on the values found from the results of three networks that have polymerized for 2 h and defined as the mean values $\pm$ two standard deviations of this parameter set. The analysis is based on $5\cdot 10^4$ model runs, which is five times the minimum advised for a model with eight parameters (Saltelli 2002).

## Results

In this section, results are presented to illustrate the performance of the model. Representative results are shown for one sample and discussed in detail. Parameter values of multiple samples are shown to illustrate the variation between samples. The model is first used to describe the first LAOS sequence and the recovery that takes place during the following 2 h. Subsequently, the parameter set obtained is used to predict the outcome of the second LAOS sequence. Finally, the model is used to describe the response for networks that have polymerized for 1 h instead of 2 h, to study the influence of the presence of cross-links created by fXIIIa.

### Describing LAOS results

**Fig. 7:**
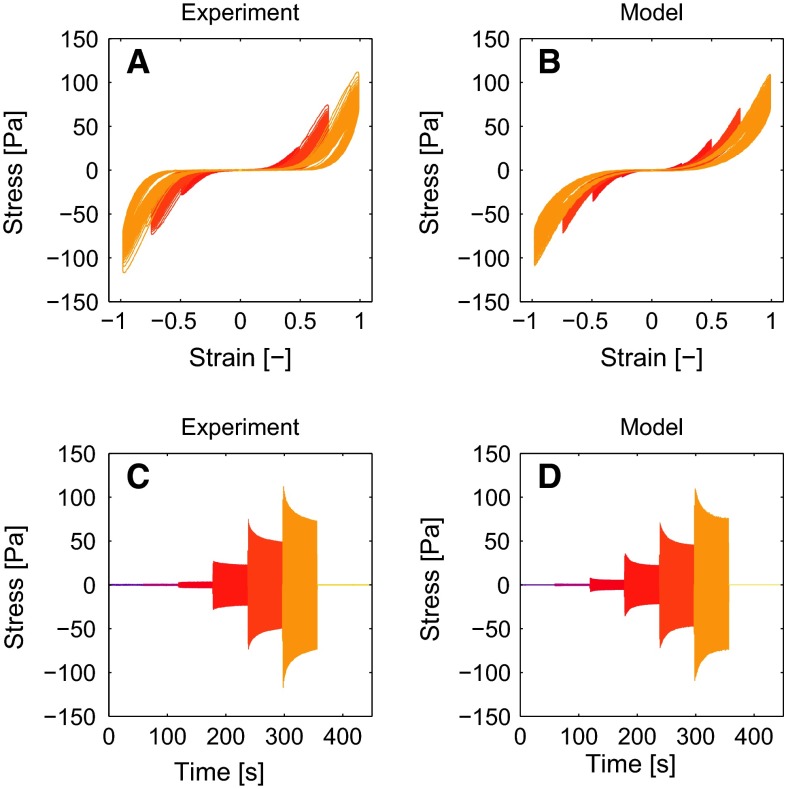
Experimental results of the first LAOS sequence (**a**, **c**) and the corresponding model description (**b**, **d**). Both the Lissajous-Bowditch plots (**a**, **b**) and the stress in time (**c**, **d**) show that the model captures the nonlinear viscoelastic behavior of the fibrin networks that have polymerized for 2 h

The experimental and numerical results for the LAOS sequence are shown in Fig. 7 as Lissajous-Bowditch plots (panel A,B) and for the stress in time (panel C,D). The three nonlinear features observed in the experimental results, being softening, strain stiffening and nonlinear viscous dissipation, are all described accurately by the model. The maximal stress values during a deformation cycle agree well, including the softening effect that occurs over multiple deformation cycles, also visible from the NSP during the LAOS sequence already shown in Fig. 3a. The nonlinear viscous dissipation is present in the model and agrees qualitatively with the experimental result, although there is room for improvement.

**Fig. 8:**
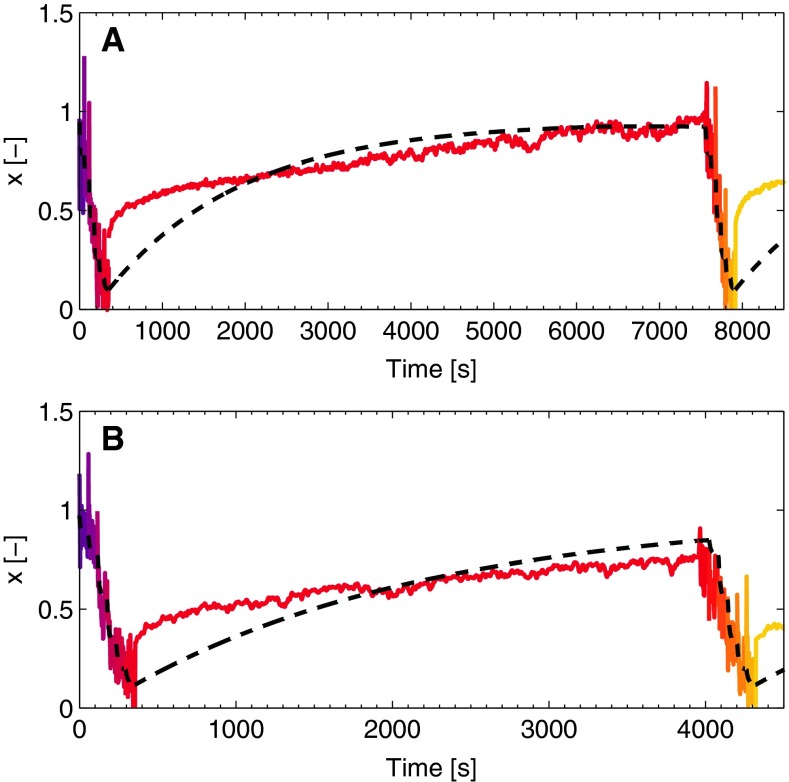
The NSP, *x*, in time illustrates the increasing stiffness following the LAOS deformation. Experimental results are shown in *colors*, with the model description as *dashed lines*. Results are shown for networks that polymerized for 2 h (**a**) and 1 h (**b**)

After the LAOS sequence, a SAOS deformation is imposed for 2 h to be able to observe the recovery of the network during this period. The results of this measurement are shown in terms of the NSP, $x$, in Fig. 8a. Both the experimentally and numerically determined $x$ increase in time. The model describes an exponential increase, while experimentally, a fast initial rise is followed by a slower increase. A more advanced kinetic equation could improve this but this has no priority for this study.

### Predicting LAOS results

**Fig. 9:**
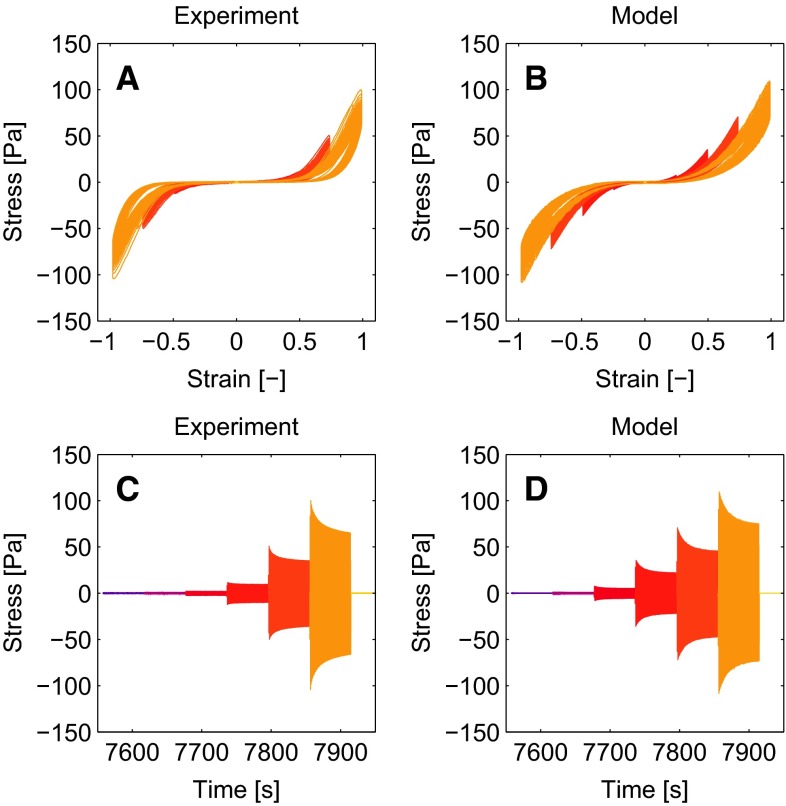
Experimental (**a**, **c**) results of the second LAOS sequence. The model results (**b**, **d**) are obtained using the parameters obtained from a model fit to the first LAOS sequence. This shows that the model can describe the second LAOS sequence using the same parameters that describe the first LAOS sequence

The parameter set found by fitting the model to the first LAOS sequence and the subsequent recovery, as described in the previous section, is used to predict the outcome of the second LAOS sequence. The results shown in Fig. 9 show that the stress during the second LAOS sequence is qualitatively the same as during the first sequence, but the maximal stress values during the cycles are slightly lower. The model describe this behavior well, but overestimates the maximal stress values.

### Influence of fXIIIa

To study the influence of cross-links created by fXIIIa, the same LAOS sequence as used before is applied to networks that have polymerized for 1 h instead of 2 h.

**Fig. 10:**
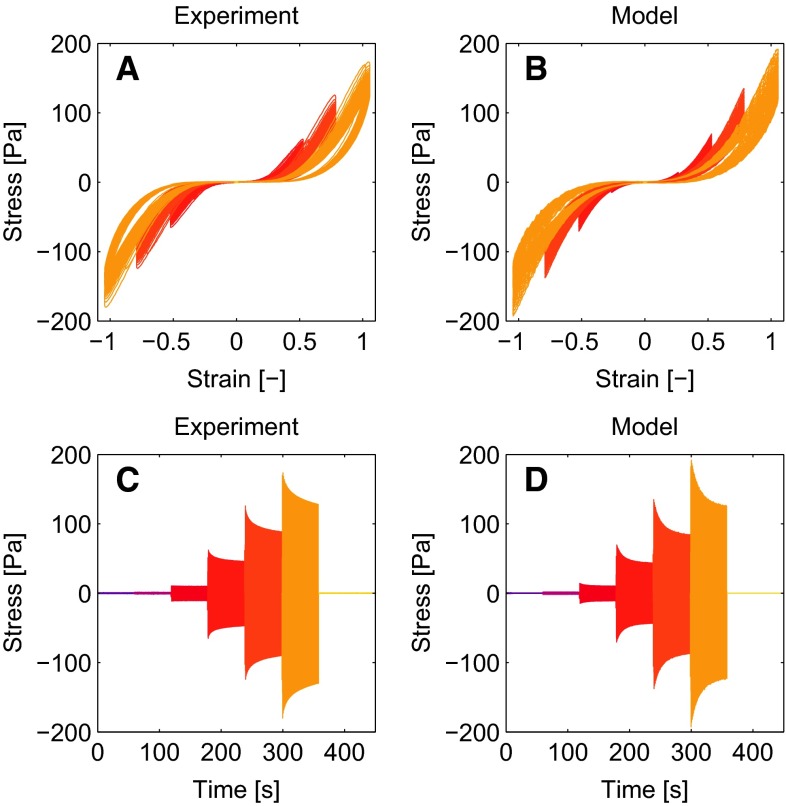
Results of a fibrin network that has polymerized for 1 h instead of two show the influences of cross-links created by fXIIIa. Experimental results (**a**, **c**) show that these networks strain stiffen more than their counterparts which is well described by the model (**b**, **d**)

Qualitatively, the results are the same as for the networks that polymerized for 2 h, and the model describes this well (Fig. 10). A difference is that the network that has polymerized for 1 h reaches a higher stress during the LAOS sequence (170 vs. 110 Pa), while the difference between the low-strain modulus before the LAOS sequence is smaller (11.6 vs. 8.4 Pa). The model overestimates the maximal stress for the largest strain amplitude by 11 $\%$, but the agreement is better for the lower strains, e.g., 7 $\%$ for a strain amplitude of 0.75.

The recovery after the LAOS sequence, also visible in the network that has polymerized for 1 h, shows similar behavior as discussed before but increases faster (Fig. 8b).

**Fig. 11:**
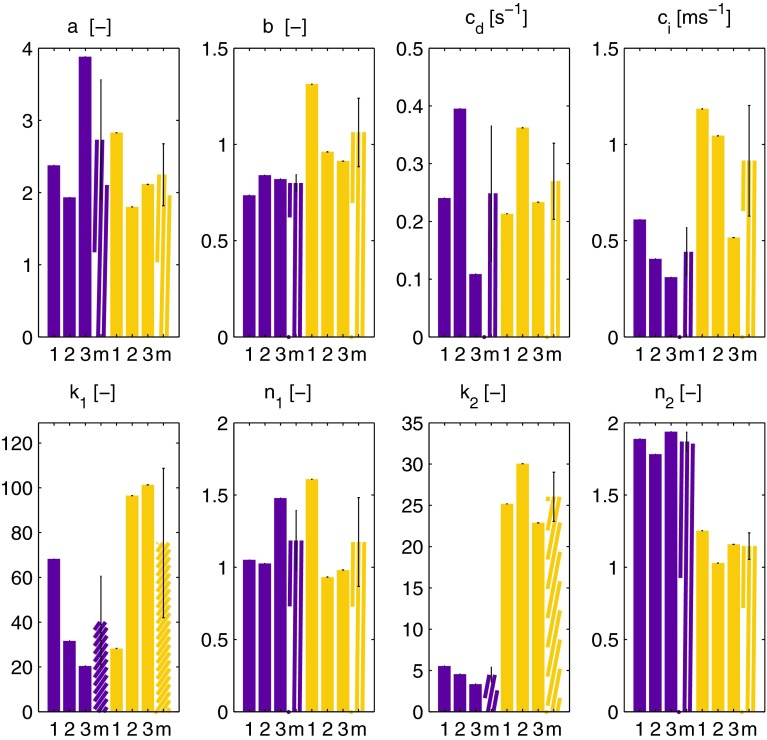
Parameter values obtained by fitting the model to experimental results for networks that have polymerized for 2 (*purple*) or 1 (*yellow*) h. Results of three independent measurements are shown per condition (1,2,3), together with the mean value (m). The *errorbar* indicates the standard deviation. The parameter values illustrate that the networks that polymerized 1 h show more strain stiffening ($k_1$, $n_1$), faster recovery after the LAOS sequence ($c_1$) and more viscous dissipation ($k_2$, $n_2$) than the networks that polymerized 2 h

The observations that the networks that have polymerized for 1 h show more strain stiffening and recover faster also follows from the parameter values found by the model. Figure 11 shows the values for the eight parameters of the model for the networks that polymerized for 2 h (purple) and 1 h (yellow), for three samples of each condition and the mean value with standard deviation. The parameters that describe the strain stiffening are $k_1$ and $n_1$. Although there is considerable variation between samples it is clear that the values of $k_1$ are higher for the networks that polymerized for 1 h. An exception to this is sample $1$, which has a relatively low value of $k_1$, but this is balanced by a high value for $n_1$, which also implies more strain stiffening.

The faster increase after the LAOS sequence is described by the parameter $c_i$. This parameter indeed has a higher value for the networks that polymerized for 1 h, indicating that the they recover faster from the LAOS sequence.

A third difference that is visible from the parameter values is the nonlinear viscous dissipation, described by $k_2$ and $n_2$. The networks that polymerized for 2 h, have lower values for $k_2$ (4.5 vs. 26) and higher values for $n_2$ (1.9 vs. 1.1). The result is that the viscous contribution increases more with increasing strain for the networks that have polymerized for 1 h, which is not directly visible from the Lissajous–Bowditch plots.

### Sensitivity analysis

**Fig. 12:**
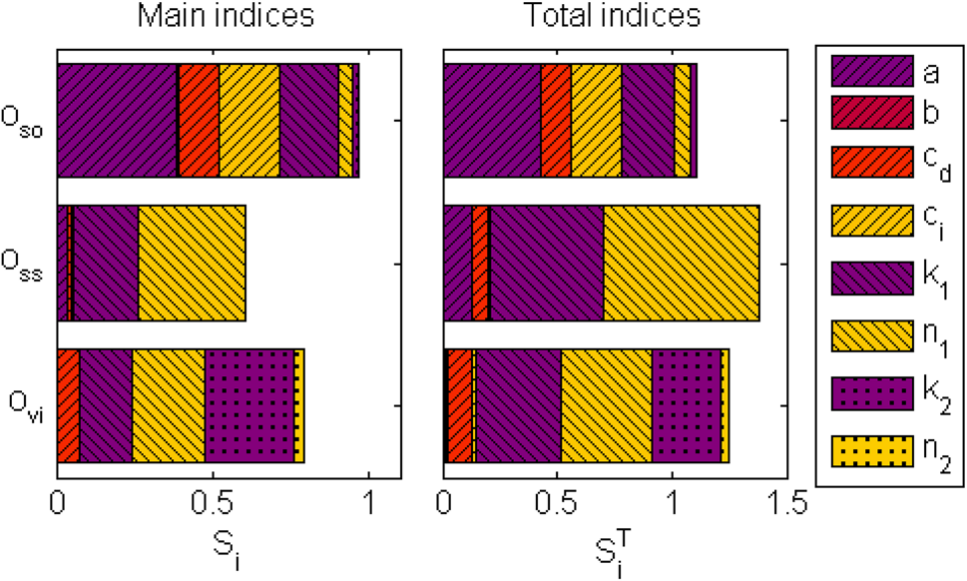
The three outputs used for the sensitivity analysis quantify the softening $O_\mathrm{so}$, strain stiffening $O_\mathrm{ss}$ and viscous $O_\mathrm{vi}$ effects. The main index, $S_i$, shows the contribution of a parameter to the variance of an output, while the total index, $S_i^T$ illustrates the higher-order contributions interactions with other parameters

A sensitivity analysis is performed to assess how the parameters influence the output uncertainty of the three defined outputs. For every output, the contribution of the parameters to the variance of the outputs is determined. These contributions are shown as a main effect, $S_i$ and a total effect, $S_i^T$ (Fig. 12).

The variance of the output related to softening, $O_\mathrm{so}$, is to a large extent determined by the parameters $a$, $c_d$ and $c_i$, as shown by their large contributions of both the main and total indices (Fig. 12). This is reasonable because these parameters are used to describe the NSP ($x$) that governs the softening behavior. However, the parameter that is involved in strain stiffening, $k_1$, also has a relatively large contribution to the softening.

As expected, the variance of the output for the strain stiffening behavior, $O_\mathrm{ss}$, is dominated by the parameters that describe stiffening, $k_1$ and $n_1$. The sum of the main indices is less than one, which is an indication that higher-order effects contribute significantly to the total variance (Huberts et al. 2014). This is also shown by the relatively large values of the total indices in comparison with the main indices.

The variance of the output related to the nonlinear viscous dissipation, $O_\mathrm{vi}$, is for a large part determined by the parameter $k_2$ that describes this behavior, but also for a large portion by $k_1$, and $n_1$ that describe the strain stiffening behavior.

## Discussion

The constitutive model proposed in this study describes the behavior of the fibrin networks during a LAOS deformation. Using the Lissajous–Bowditch plots, three nonlinear viscoelastic features have been distinguished and are subsequently incorporated in a Kelvin–Voigt model. Although the features are modeled in a phenomenological way, it is possible to relate them to structural changes in the network. The softening observed during the LAOS sequence originates from the semi-permanent elongation of fibers due to the imposed deformation (Münster et al. 2013). During a repeating deformation, fibers become longer in the rest state, leading to a lower stiffness when the same deformation is reached again. This process explains why the NSP, that relates the deformation history to the stiffness of the network, decreases during an increasing strain. Fibers become longer during the deformation but this effect disappears when all fibers have adapted to the current strain, which explains why the NSP levels off to a values corresponding with that strain. Besides, it might be possible that breakage of connections between fibers occurs, which can also lead to an exponential decrease of the stiffness (Abhilash et al. 2012). Both lengthening of fibers and breakage of connections is inhibited by the cross-links created by fXIIIa (Münster et al. 2013). This can explain why the parameters $b$ and $c_d$ that describe the decrease of the NSP and the corresponding time scale, respectively, have slightly higher values for the samples that polymerized for 1 h instead of two.

During the SAOS experiment that follows the LAOS sequence,the stiffness of the network increases. Experimentally, two time scales are observed. A fast initial rise in stiffness is followed by a slower increase for a longer time. The latter is attributed to the creation of cross-links by fXIIIa (Ryan et al. 1999; Lorand 2005) while the former might be caused by fibers that have the opportunity to become shorter again due to the smaller deformation. Recently, it has been shown that the fibrin network is a dynamic structure and that parts of fibers rearrange within the network (Chernysh et al. 2012). This remodeling might take place and can explain the increase in stiffness, and also why this happens on a slower time scale when fXIIIa is present since the creation of cross-links decreases the amount of rearrangements (Chernysh et al. 2012). Both phenomena could be incorporated in more detail using more structural information but this is considered outside the scope of the current study.

Fibrin networks show a remarkable amount of strain stiffening (Storm et al. 2005; Onck et al. 2005; Brown et al. 2009; Piechocka et al. 2010; Weigandt et al. 2011) which is modeled here as an increase of the shear modulus as function of the strain. Several mechanisms have been suggested to explain the origin of this stiffening, including entropic stretching (Storm et al. 2005), non-affine deformation of fibers (Onck et al. 2005) and protein unfolding (Brown et al. 2009). It is expected that a combination of these mechanisms takes place (Piechocka et al. 2010; Weigandt et al. 2011).

The relation between mechanical and structural properties of the fibrin network has been studied under various conditions (Brown and Barker 2014). The fibers within the network align and subsequently stretch due to an increasing deformation (Brown et al. 2009). Using a computational analysis, it has been shown that the individual fiber properties and their alignment can be related to the stiffness of the network (Kim et al. 2011). Further structural clues can be obtained by the behavior of the fibrin network under compression, where the network first softens due to buckling and bending of fibers, followed by stiffening due to a densification of the network at larger compressions (Kim et al. 2014). Such structural information can be used to extent the model and describe the mechanical properties of the fibrin network based on its structure. However, the current phenomenological description already shows satisfying results and more complexity is not wanted in view of the desired application of advanced numerical simulations of blood clot formation (Storti et al. 2014).

The constitutive model is used to describe the behavior of the fibrin network during a LAOS deformation and the increasing stiffness observed afterward. The results show that during these 2 h, a relatively fast increase of the stiffness is followed by a more gradual increase. These two effects, attributed to fiber remodeling and creation of cross-links, respectively, are not described by the model explicitly, since a single time constant is used. This explains why the description of the stiffness during this phase is relatively poor compared with the description during the LAOS deformation. However, the model is such that it can easily be extended to better describe this phase, but this has not been done here to focus on the nonlinear mechanical behavior of the fibrin network during the LAOS sequence.

The model developed contains eight fitting parameters. The values for these parameters are found in three separate fitting procedures, using the data of the entire LAOS deformation. Therefore, an obtained parameter set describes the LAOS experiment as a whole and is not necessarily the best parameter set to describe a single deformation cycle. However, as shown in Fig. 4b, the agreement for a single cycle is reasonably well.

A sensitivity analysis has been performed to study the influence of variations in parameter values. For three outputs, related to the three observed nonlinear features, the contribution of every parameter to the variance of this output is determined. The expectation that the parameters used to model one of the effects have the largest influence on the output of this effect is confirmed. An example is that the parameter $k_1$ and $n_1$, used to describe strain stiffening, have the largest contribution to the variance of the output $O_\mathrm{ss}$. However, it is also found that interactions exist between the three effects. An example is that the parameters for the strain stiffening behavior have a large contribution to the output $O_\mathrm{vi}$ that quantifies the nonlinear viscous dissipation. Two parameters, $b$ and $n_2$, have small contributions to all the outputs as shown by small total indices (Fig. 12). This is an indication that they could be fixed within their uncertainty interval.

In this study, a LAOS deformation is used to study the nonlinear viscoelastic properties of fibrin. Other protocols could have been used, such as strain ramps (Schmoller et al. 2010) or prestress (Piechocka et al. 2010). An advantage of the LAOS deformation used here is that it mimics the large oscillatory deformation occurring in blood vessels. Furthermore, the analysis in terms of Lissajous–Bowditch plots applied here illustrates that this method can be used to distinguish various nonlinear effects that occur. The model is developed such that it can describe any arbitrary deformation history because it uses the time-dependent strain as an input. Therefore, the model could be used to describe other deformation protocols as mentioned above as well.

The model developed works well in describing the nonlinear behavior of the fibrin networks. The model is flexible and can easily be adapted to describe the mechanical behavior of other biopolymers that show similar behavior such as collagen (Kurniawan et al. 2012; Münster et al. 2013), hagfish slime (Ewoldt et al. 2011), keratin filaments (Ma et al. 1999), gluten gel (Ng et al. 2011) and also soft tissues such as skin (Lamers et al. 2013). Although the mechanical behavior of these materials is complex, the model is deliberately kept simple to make it suitable for numerical simulations of blood clot formation (Storti et al. 2014) and to combine it with a model for fibrin network maturation (van Kempen et al. 2014).

## Conclusion

A constitutive model has been developed to describe the time-dependent, nonlinear viscoelastic behavior of fibrin networks. The model is developed using experimental results of a LAOS deformation and describes the observed softening, strain stiffening and increasing viscous dissipation that occur during multiple deformation cycles. Furthermore, an increasing stiffness that takes place after the LAOS deformation is captured. The model is able to describe all these features, and its generality makes it suitable to be applied to other materials showing similar behavior.
